# Representation Alignment with Hybrid Perturbation for Semi-Supervised Medical Image Segmentation

**DOI:** 10.3390/s26206664

**Published:** 2026-10-21

**Authors:** Huaikun Zhang, Shiqiang Du, Jing Lian

**Affiliations:** 1 School of Mathematics and Computer Science, Northwest Minzu University, Lanzhou 730030, China; shiqiangdu@xbmu.edu.cn; 2 School of Electronic and Information Engineering, Lanzhou Jiaotong University, Lanzhou 730070, China; lian322scc@163.com

**Keywords:** medical image segmentation, semi-supervised learning, hybrid perturbation, cross teaching, representation alignment

## Abstract

**Highlights:**

**What are the main findings?**

- RAHP integrates network-, image-, and feature-level perturbations to construct complementary training views for semi-supervised medical image segmentation.
- Region-aware patch alignment stabilizes intermediate representations and reduces perturbation-induced representation inconsistency.

**What are the implications of the main findings?**

- Patch-level representation alignment provides complementary supervision beyond conventional prediction-level consistency.
- RAHP improves the utilization of unlabeled medical images and supports stable segmentation performance under limited annotations.

**Abstract:**

Given the scarcity of labeled data and the high cost of annotation in medical imaging, semi-supervised learning (SSL) has become a promising approach for semantic segmentation tasks. In this paper, we propose RAHP, a representation stabilization framework for semi-supervised medical image segmentation, which addresses perturbation-induced representation inconsistency by integrating hybrid perturbation learning with explicit intermediate representation alignment. Our method adopts a cross-teaching architecture in which two sub-networks guide each other through pseudo-labeling to ensure stable training. To improve consistency across diverse perturbed views, we employ a hybrid perturbation strategy to generate complementary training views. Rather than relying only on prediction-level consistency between these perturbed views, we further introduce a Region-aware Patch Alignment Loss (RPAL) to explicitly align spatially corresponding decoder representations at the patch level. By combining prediction-level supervision with region-aware representation alignment, RAHP constrains not only the output predictions but also the intermediate semantic representations induced by heterogeneous perturbations. Experiments on three public datasets, including ACDC, Synapse, and MMWHS, show that RAHP achieves favorable mean performance compared with the evaluated semi-supervised methods under the reported limited-label settings.

## 1. Introduction

Medical image segmentation is a fundamental and critical task in computer-aided diagnosis (CAD), aiming to accurately identify target organs, tissues, or lesion areas at the pixel level [1,2]. With the rapid development of deep learning, fully supervised medical image segmentation methods have achieved remarkable progress by leveraging large-scale pixel-level annotations [3,4,5]. However, obtaining dense pixel-level annotations remains costly and time-consuming, especially for cardiac and multi-organ images that present complex anatomy, weak boundaries, and large inter-subject variations. These challenges greatly limit the practical applicability of fully supervised segmentation methods in clinical scenarios [6,7]. Therefore, reducing the dependency on extensive annotations while maintaining segmentation performance has become an important research direction. Semi-supervised learning approaches have consequently attracted increasing attention by exploiting large amounts of unlabeled data together with limited labeled samples [8,9].

Among existing approaches, consistency regularization has been widely adopted due to its effectiveness and conceptual simplicity. By encouraging prediction agreement under different perturbations, consistency-based methods enable supervision to be transferred from labeled to unlabeled samples in a stable manner [10,11,12]. To strengthen consistency learning, early methods focused on input-level perturbations with weak-to-strong augmentation strategies to enforce reliable pseudo-label supervision [13]. More recent methods have introduced strong perturbations and multi-view training strategies such as cross-teaching and mutual learning frameworks [14,15], as well as hybrid perturbation schemes combining multiple input- and feature-level perturbation views [16,17,18]. These methods enforce prediction agreement across different networks or different transformed views of the same input. Feature-level perturbations have also been explored to further increase representation diversity, with recent works incorporating probabilistic prototypes and edge-aware contrastive representations for improved robustness [19,20]. Although these strategies improve robustness, strong perturbations inevitably introduce additional training instability, particularly when pseudo labels are still inaccurate at early stages. Representative methods illustrate different ways of constructing diverse consistency views. FixMatch [13] establishes weak-to-strong input consistency by using predictions from weakly augmented samples to supervise strongly augmented counterparts. BCP [21] introduces bidirectional copy-paste between labeled and unlabeled samples within a Mean Teacher framework, whereas UniMatch [18] further expands the perturbation space by combining dual strong image views with an auxiliary feature perturbation stream. These methods demonstrate the effectiveness of increasing view diversity for semi-supervised learning. However, their primary consistency supervision is imposed on the resulting predictions, while the local intermediate representations induced by different perturbation views are not explicitly aligned.

This limitation becomes particularly relevant under strong perturbations, where features corresponding to the same anatomical region may become scattered in the embedding space, thereby weakening regional semantic coherence. This issue is especially critical for medical images, where anatomical structures are spatially continuous and exhibit clear structural priors. When the representation space is not explicitly regularized, prediction discrepancies induced by perturbations may further affect the stability of subsequent pseudo-supervised learning.

To address the above limitation, we propose RAHP, a representation stabilization framework that explicitly reduces perturbation-induced representation inconsistency in semi-supervised medical image segmentation. Instead of introducing another perturbation generation strategy, RAHP focuses on maintaining stable semantic representations under diverse perturbations. Within RAHP, the hybrid perturbation pathway is used to construct complementary training views from the unlabeled data. On top of these diverse views, a Region-aware Patch Alignment Loss (RPAL) is introduced to explicitly align intermediate representations between the two sub-networks at the patch level. In this way, perturbation diversity provides the training views, whereas RPAL supplies an additional representation-level constraint beyond conventional prediction consistency. By enforcing local representation consistency for corresponding anatomical regions, RPAL stabilizes the representation space under strong perturbations and limits perturbation-induced cross-view discrepancies during pseudo-supervised training. The proposed framework has two complementary components. Hybrid perturbations provide complementary supervision across multiple levels, which improves the generalization ability of the model under limited annotations. RPAL introduces an explicit constraint in the feature space, which enhances regional discrimination and leads to more stable boundary prediction. Our experiments suggest that patch-level inconsistency under strong perturbations may contribute to representation inconsistency in semi-supervised medical image segmentation, which is insufficiently considered by existing consistency-based frameworks. Our main contributions are as follows:

- We propose RAHP, a representation stabilization framework for semi-supervised medical image segmentation that augments dual-network cross teaching with explicit representation-level regularization under heterogeneous perturbation views.
- We design a Region-aware Patch Alignment Loss (RPAL), which aligns spatially corresponding decoder patches through learned region-aware weights and provides an explicit local representation constraint complementary to prediction-level pseudo-label supervision.
- We evaluate the proposed method on three public medical image segmentation datasets, where it achieves favorable mean performance compared with the evaluated semi-supervised methods under limited-label settings.

## 2. Related Work

### 2.1. Medical Image Segmentation

Medical image segmentation involves classifying and identifying medical images on a pixel-by-pixel basis, which can significantly aid clinicians in diagnosing diseases. Deep learning-based medical image segmentation can be categorized into two types based on the amount of labeled data used: fully supervised segmentation methods and semi-supervised segmentation methods.

For fully supervised segmentation methods, FCN [22] and U-Net [4] are considered foundational frameworks for fully convolutional network segmentation. Subsequent CNN-based methods have continuously improved performance by increasing the receptive field [23,24] or incorporating attention mechanisms [25,26]. With the rapid development of transformers in computer vision, many researchers have proposed transformer-based or CNN-transformer hybrid methods [27,28,29] that leverage the transformer’s ability to capture long-range dependencies, thereby further improving segmentation performance.

Despite the impressive results achieved by fully supervised algorithms, their performance is limited by the scarcity of labeled medical images and the high cost of annotations. Consequently, more semi-supervised medical image segmentation methods have emerged. Among these semi-supervised methods, the main approaches include consistency regularization [30,31] and pseudo labeling [32,33]. Additionally, some research focuses on adversarial learning methods [34,35] and contrastive learning methods [36,37].

### 2.2. Consistency Regularization-Based SSL

In recent years, consistency regularization has become a popular approach in semi-supervised learning. This method assumes that the predictions for an unlabeled example should remain invariant under different forms of perturbations. Consistency regularization was first proposed by [38], followed by methods such as the Pi-model [10], Mean Teacher [12], and co-training [39], most of which focus on network-level perturbations. For instance, Mean Teacher enforces consistency between predictions from a student and a teacher network.

Recently, beyond network-level perturbations, image-level perturbations, particularly strong augmentations, have also been widely explored. Cutout [40] and CutMix [41] enhance the diversity of training data by occluding parts of an image or mixing regions from different images, thereby significantly improving the robustness and generalization capabilities of deep learning models. FixMatch [13] introduced the idea of injecting strong perturbations into unlabeled images and supervising the training process using predictions from weakly perturbed images. Subsequent methods like FlexMatch [42] and FreeMatch [43] proposed dynamically adjusting the pseudo-label threshold based on the current learning state and class-specific conditions, replacing the fixed threshold setting in FixMatch. The recent UniMatch [18] further expands the perturbation space by incorporating feature-level perturbations and employing a dual-path strong perturbation strategy, thereby enhancing network performance. BCP [21] introduced a novel image-level perturbation strategy by bidirectionally copying and pasting labeled and unlabeled data within a simple mean teacher architecture. More recently, promptable foundation models such as the Segment Anything Model (SAM) have been introduced into semi-supervised medical image segmentation. SemiSAM+ [8] employs pretrained promptable foundation models as generalist models to provide additional supervision for a task-specific specialist model under limited annotations. Konwer et al. [9] further explore SAM-assisted semi-supervised segmentation through efficient prompting and preference optimization. More recent studies have extended this direction with more explicit prompt-generation and cross-model interaction mechanisms. CPAC-SAM [44] introduces SAM-driven cross-prompting together with adaptive prompt sampling and prompt-consistency learning, while AutoPromptSeg [45] employs uncertainty-guided automatic prompt generation to exploit SAM for semi-supervised segmentation. These studies demonstrate the growing potential of pretrained foundation models to support annotation-efficient medical image segmentation through prompt-based supervision and knowledge transfer.

Collectively, FixMatch, BCP, and UniMatch improve semi-supervised learning by constructing diverse input- or feature-level views and enforcing consistency primarily at the prediction level. RAHP follows the same general perturbation-based paradigm but addresses a complementary aspect of the learning process: explicit regularization of intermediate representations across heterogeneous views. Specifically, RPAL aligns spatially corresponding decoder representations at the patch level and assigns adaptive region weights during alignment. Thus, the perturbation mechanisms are used to construct diverse views, while RPAL provides an additional local representation-level constraint beyond prediction-level consistency.

## 3. Materials and Methods

For a semi-supervised semantic segmentation task, given an input image $x\in R^{H\times W}$, our goal is to learn a segmentation network capable of assigning each pixel in the input image x to its corresponding K-class semantic segmentation label. The training dataset consists of two subsets: $D^{l}={\left\{x_{i}^{l},y_{i}^{l}\right\}}_{i=1}^{N}$, which comprises a limited amount of labeled data, and $D^{u}={\left\{x_{j}^{u}\right\}}_{j=1}^{M}$, which consists of a substantial quantity of unlabeled data, with *N* << *M*.

In this paper, we present RAHP, a semi-supervised segmentation framework that combines perturbation-based consistency learning with intermediate representation regularization. RAHP integrates network-level, image-level, and feature-level perturbations, together with a representation alignment module that enforces semantic consistency between strongly perturbed features across networks. This design improves the diversity of consistency constraints while explicitly regularizing intermediate representations, enabling more stable feature learning under limited supervision. In the following sections, we detail the hybrid perturbation strategy, the representation alignment loss, and the overall training mechanism.

### 3.1. Hybrid Perturbation Strategy 

**Figure 1:**
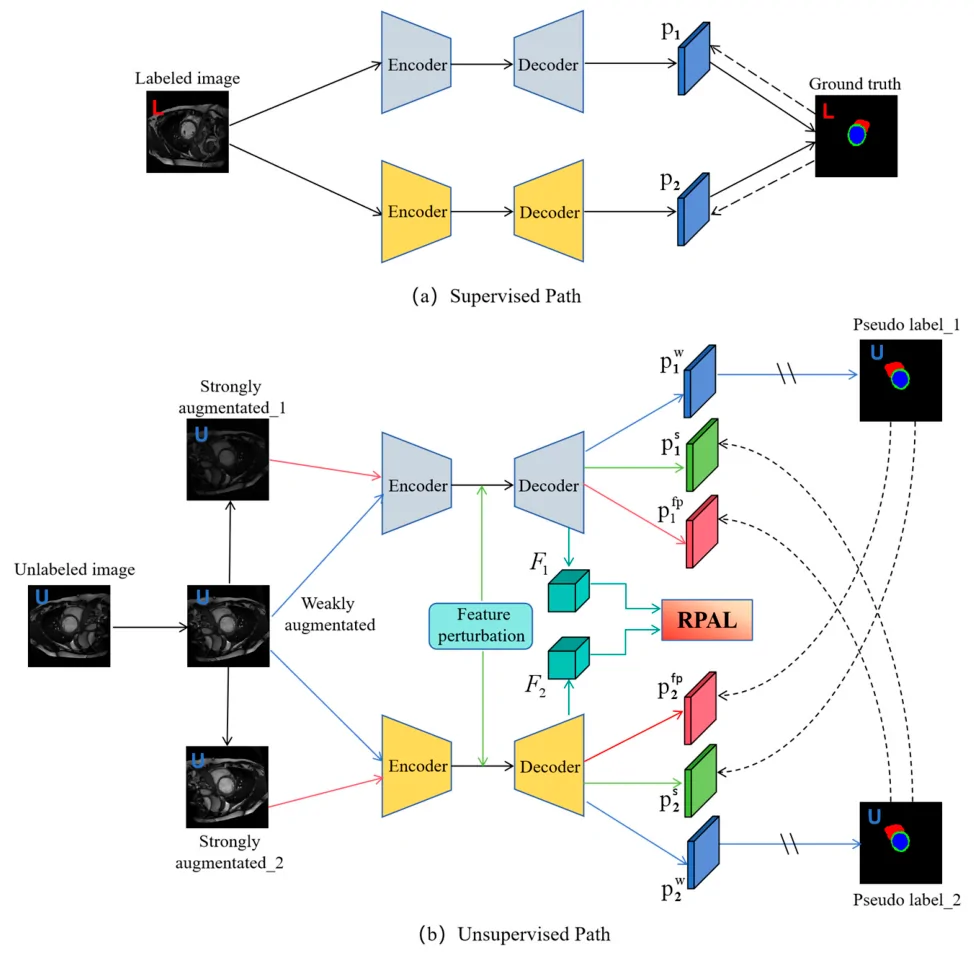
Overall framework of RAHP. Labeled samples are optimized through a supervised path, while unlabeled samples are trained via consistency learning under hybrid perturbations. Predictions from weakly perturbed inputs provide pseudo supervision for strongly perturbed counterparts. The RPAL module enforces region-aware alignment on intermediate decoder features, stabilizing representation learning under strong perturbations. The colors distinguish the two network branches and different prediction paths, while the dashed arrows indicate cross-supervision between predictions and pseudo-labels.

The overall structure of RAHP is shown in Figure 1. Our approach builds upon a dual-network cross-teaching framework, where two sub-networks with identical architectures but different initialization parameters are trained simultaneously. In this work, network-level perturbation does not refer to an additional stochastic transformation explicitly applied to the network parameters. Instead, it arises from the parameter diversity between the two sub-networks, which share the same architecture but are independently initialized and optimized. Consequently, the two branches follow different optimization trajectories and produce complementary predictions during training. These parameter differences constitute the network-level perturbation used in the cross-teaching framework. For the labeled data, each sub-network’s predictions are supervised using the corresponding ground truth segmentation labels. For the unlabeled data, the conventional cross-training supervision method converts the predictions of the two sub-networks into one-hot pseudo labels via the argmax operation, which are then used to supervise the other sub-network’s predictions.

Although the cross-teaching framework effectively enforces consistency between the two networks, relying solely on this mechanism is insufficient to ensure robustness against input and feature variations. To address this limitation, we employ a hybrid perturbation strategy that integrates three complementary types of perturbations: network-level perturbation, image-level perturbation, and feature-level perturbation. The combination of these perturbations enlarges the diversity of training views and provides a stronger consistency constraint. However, excessive perturbation diversity may introduce representation discrepancy between different views, motivating the proposed RPAL module. The hybrid perturbation component is used to construct heterogeneous training views, while the representation discrepancy between these views is explicitly regularized by RPAL, as described in Section 3.2.

#### 3.1.1. Training Paradigm and Loss Function

The training objective consists of a supervised segmentation loss for labeled samples and two consistency losses for unlabeled samples, including image-level and feature-level perturbation consistency. The supervised loss is computed by comparing the predictions of the two sub-networks on weakly perturbed labeled samples against their corresponding ground truth annotations, and is formulated as follows:

(1) $$p_{1}^{l,w},\;p_{2}^{l,w}={f(A}^{w}(x^{l});\;\theta_{1}),\;{f(A}^{w}(x^{l});\;\theta_{2}),$$

(2) $$y^{l,w}=A^{w}(y^{l}),$$

(3) $$L_{sup}=\frac{1}{B_{l}}\sum_{b=1}^{B_{l}}\left(l(p_{1,b}^{l,w},\;y_{b}^{l,w}\right)+l(p_{2,b}^{l,w},\;y_{b}^{l,w})),$$

where $A^{w}$ denotes weak augmentation, $B_{l}$ represents the batch size of labeled data, $y^{l,w}$ denotes the true labels of labeled data after weak perturbations, $p_{1}^{l,w}$ and $p_{2}^{l,w}$ represent the segmentation confidence maps obtained by feeding labeled data after weak perturbations into sub-networks 1 and 2, l(·,·) denotes the segmentation loss used to measure the discrepancy between the prediction and the corresponding ground-truth label.

For unlabeled data, we employ a dual-path strategy that incorporates both image-level and feature-level perturbations. Specifically, weakly augmented versions of unlabeled images are first fed into each sub-network to generate pseudo labels. Stronger augmentations are applied to the same images and passed through the sub-networks to produce perturbed segmentation predictions. These predictions are then cross-supervised by the pseudo labels obtained from the other sub-network. The unsupervised loss based on image-level perturbations is formulated as follows:

(4) $$p_{1}^{u,w},\;p_{2}^{u,w}={f(A}^{w}(x^{u});\;\theta_{1}),\;{f(A}^{w}(x^{u});\;\theta_{2}),$$

(5) $$y_{1}^{u,w},\;y_{2}^{u,w}=argmax(p_{1}^{u,w}),\;argmax(p_{2}^{u,w}),$$

(6) $$p_{1}^{u,s},\;p_{2}^{u,s}={f(A}^{s}(x^{u});\;\theta_{1}),\;{f(A}^{s}(x^{u});\;\theta_{2}),$$

(7) $$L_{u}^{img}=\frac{1}{B_{u}}\sum_{b=1}^{B_{u}}\left[𝟙(max(p_{2,b}^{u,w})\geq \tau )l(p_{1,b}^{u,s},\;y_{2,b}^{u,w}\right)+𝟙(max(p_{1,b}^{u,w})\geq \tau )l({p_{2,b}^{u,s},\;y}_{1,b}^{u,w})],$$

where $A^{s}$ denotes strong augmentation, $B_{u}$ represents the batch size of unlabeled data, τ is a predefined confidence threshold applied to exclude noisy pseudo labels, $y_{1}^{u,w}$ and $y_{2}^{u,w}$ respectively denote the pseudo labels predicted by feeding weakly perturbed unlabeled data into sub-networks 1 and 2, while $p_{1}^{u,s}$ and $p_{2}^{u,s}$ represent the segmentation confidence maps obtained by feeding strongly perturbed unlabeled data into sub-networks 1 and 2.

To broaden the perturbation space, we also perturb the features extracted from the encoder output during the forward pass of the network on unlabeled images, thereby obtaining output prediction results perturbed through feature manipulation.

(8) $$z=h(x^{u,w}),$$

(9) $${\;p}^{feat}=g(FP(z)),$$

where h(·) and g(·) respectively represent the encoder and decoder of the network, while FP denotes feature perturbation, which is implemented by randomly dropping channels from the encoder output feature maps during training. Based on this, we propose another supervision pathway for unlabeled data, namely cross-supervision of the output results based on feature perturbation.

(10) $$L_{u}^{feat}=\frac{1}{B_{u}}\sum_{b=1}^{B_{u}}\left[𝟙(max(p_{2,b}^{u,w})\geq \tau )l(p_{1,b}^{feat},\;y_{2,b}^{u,w}\right)+𝟙(max(p_{1,b}^{u,w})\geq \tau )l({p_{2,b}^{feat},\;y}_{1,b}^{u,w})],$$

Overall, the objective associated with hybrid perturbation learning consists of the supervised segmentation loss and two unsupervised consistency losses derived from image-level and feature-level perturbations:

(11) $$L_{hybrid}=L_{sup}+\lambda_{img}L_{u}^{img}+{\lambda_{feat}L}_{u}^{feat},$$

Here, $\lambda_{img}$ and $\lambda_{feat}$ control the contributions of the image-level and feature-level consistency losses, respectively. The supervised loss $L_{sup}$ combines cross-entropy loss and Dice loss, whereas the two unsupervised consistency terms use Dice loss.

#### 3.1.2. Perturbation Types and Configurations

In this section, we introduce various methods for selecting and configuring perturbations in detail. Drawing inspiration from FixMatch, we embrace a progressive augmentation strategy for input images, transitioning from weak to strong perturbations. For all unlabeled images, we apply weak augmentations such as random cropping, flipping, and rotation. For strong augmentation selection, we primarily choose strong perturbations based on image-level considerations, such as Color Jitter, blur, and CutMix. Initially, we apply random Color Jitter to the weakly augmented images, fine-tuning parameters like brightness, contrast, saturation, and hue. Following this, we introduce random blur to the Color Jitter-enhanced images, comprising Gaussian blur, average blur, and median blur, with the specific blur method selected randomly for each augmentation iteration. Finally, CutMix is applied to further diversify the strongly augmented views. 

Leveraging the cross-teaching paradigm, we independently subject the weakly augmented images $\left(x_{1}^{w},\;x_{2}^{w}\right)$ to strong augmentation $A^{s}$ for each of the two sub-networks, yielding $\left(x_{1}^{s},\;x_{2}^{s}\right)$. Given the stochastic nature of these transformations, the resulting strongly augmented images $x_{1}^{s}$ and $x_{2}^{s}$ exhibit distinct characteristics. Each unlabeled image is first subjected to a shared weak spatial transformation, including cropping, flipping, and rotation. The resulting weakly augmented image is used as the common spatial reference for both sub-networks. Two strong views are then generated independently from this shared weak view using appearance-level perturbations, including color jitter and random blur. These transformations modify image appearance but do not change spatial coordinates or anatomical locations. CutMix is subsequently applied to the two strong views using independently sampled masks. For each branch, the same CutMix mask is applied to the input image and its corresponding pseudo-label map, ensuring spatial consistency between the prediction and supervision. Importantly, RPAL is computed from the decoder features of the strong views before CutMix is applied. Therefore, the independently sampled CutMix masks affect only the prediction-level consistency pathway and do not alter the patch correspondence used by RPAL. The channel dropout operation is independently applied to the two sub-networks to maintain perturbation diversity. Details on the perturbation methods and parameter configurations are given in Table 1.

### 3.2. Region-Aware Patch Alignment Loss (RPAL)

**Figure 2:**
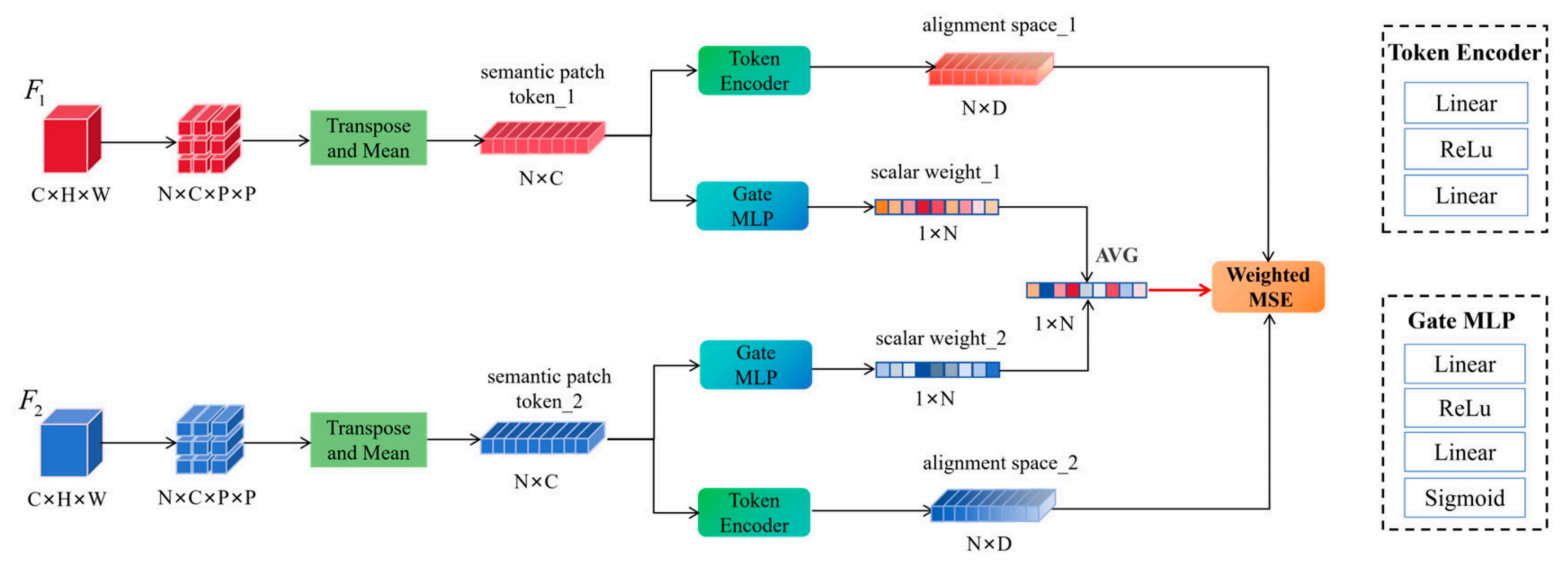
Illustration of the proposed RPAL. Colors are used to distinguish different processing modules, and arrows indicate the information flow through the RPAL module.

Although the hybrid perturbation strategy improves consistency across different input and feature transformations, the consistency constraints are still imposed primarily at the prediction level. In practice, segmentation errors often arise when structurally important regions are perturbed, as the networks fail to maintain coherent representations across different views. Prediction-level consistency does not directly constrain discrepancies in intermediate feature representations, and such discrepancies may lead to additional prediction variation across perturbed views. To address this limitation, we propose RPAL, which explicitly aligns the latent patch-level features of the two sub-networks while adaptively emphasizing more informative local regions in the feature space, as illustrated in Figure 2.

In RPAL, we operate on intermediate decoder features that retain both rich semantic information and sufficient spatial detail, rather than on the final high-resolution decoder output. Specifically, we select the decoder feature maps produced at an effective output stride of 4, meaning that the feature resolution is one quarter of the input resolution and thus preserves fine structural patterns while being computationally efficient. As shown in Figure 2, the decoder features from the two sub-networks are first partitioned into a fixed grid of non-overlapping patches, and each patch is converted into a compact representation that summarizes local semantic information before region-aware weighting and feature alignment. Concretely, let $F_{1},\;F_{2}\in R^{B\times C\times H\times W}$ denote the decoder feature maps produced by the two sub-networks from the pre-CutMix strong views derived from the same weakly augmented inputs, where *B* is the batch size, *C* the number of channels, and *H* × *W* the spatial resolution. Because both branches inherit the same weak spatial transformation and the subsequent strong appearance perturbations do not modify spatial coordinates, corresponding patches are identified deterministically by their identical grid indices in the two feature maps. Thus, the *j*-th patch in one branch is aligned only with the *j*-th patch in the other branch, and both patches refer to the same spatial region of the shared weakly augmented image. Because RPAL is computed before CutMix, semantic mismatches introduced by independently sampled CutMix masks do not enter the RPAL computation. Therefore, no mismatch-specific masking or weighting is required in the RPAL loss. First, each feature map is partitioned into a grid of non-overlapping patches of spatial size p×p, resulting in N=(H/p)·(W/p) patches per image. We then transpose and reshape to obtain the patch tensor array of shape:

(12) $$P_{i}\in R^{B\times N\times C\times p\times p},\;i\in \{1,\;2\},$$

where $P_{i,b,j}$ is the *j*-th patch (a C×p×p tensor) of the *b*-th sample from branch *i*. To obtain a compact descriptor for each patch, we apply channel-preserving spatial averaging:

(13) $$m_{i,b,j}=\frac{1}{p^{2}}\sum_{u=1}^{p}\sum_{v=1}^{p}P_{i,b,j}[:,u,v]\in R^{C},$$

This operation compresses each patch into a semantic patch token that summarizes its regional semantic content while being invariant to small local variations. Each semantic patch token is subsequently projected into an alignment space through a shared nonlinear mapping $\phi :R^{C}\to R^{D}$.

(14) $$z_{i,b,j}=\phi (m_{i,b,j})\in R^{D},$$

Here, ϕ is realized by a lightweight token encoder that captures channel correlations and provides a compact token representation. In parallel, a gating network assigns each patch a scalar weight that controls its contribution to feature alignment:

(15) $$\alpha_{i,b,j}=\sigma (\psi (m_{i,b,j}))\in (0,1),$$

where σ denotes the sigmoid function. Higher weights cause the corresponding patches to contribute more strongly to the alignment loss, whereas lower weights reduce their contribution. The detailed architecture and dimensional configuration of the token encoder and gating branch are provided in Section 4.2. To ensure symmetry between the two sub-networks, we compute the final patch importance as the average of their respective gates:

(16) $$w_{b,j}=\frac{\alpha_{1,b,j}+\alpha_{2,b,j}}{2},$$

Before measuring alignment, we normalize the alignment spaces to unit length in order to emphasize angular similarity and reduce scale sensitivity:

(17) $${ẑ}_{i,b,j}=\frac{z_{i,b,j}}{{\left\|z_{i,b,j}\right\|}_{2}+\epsilon },$$

The discrepancy between corresponding tokens from the two sub-networks is then computed as the squared Euclidean distance:

(18) $$\delta_{b,j}={\left\|{ẑ}_{1,b,j}-{ẑ}_{2,b,j}\right\|}_{2}^{2},$$

Since the embeddings are normalized, this squared Euclidean distance is proportional to cosine dissimilarity, making it a natural choice for comparing directional similarity in the feature space. Finally, RPAL aggregates patch-wise discrepancies using the region-aware weights and normalizes them within each image:

(19) $$L_{rpal}=\frac{1}{B}\sum_{b=1}^{B}\frac{\sum_{j=1}^{N}w_{b,j}\delta_{b,j}}{\sum_{j=1}^{N}w_{b,j}+\epsilon },$$

This loss encourages the two sub-networks to maintain consistent local representations in regions assigned higher alignment weights. To further clarify the role of RPAL, we next analyze how the representation discrepancy constrained by $L_{rpal}$ is related to cross-view prediction and pseudo-label supervision discrepancies.

From a regularized estimation perspective, the two pre-CutMix strong views can be regarded as different perturbation observations of the same underlying anatomical content. The consistency losses defined in Section 3.1 provide prediction-level data fitting under pseudo-label supervision, whereas $L_{rpal}$ acts as an additional structural regularizer that penalizes disagreement between corresponding local representations. To establish a connection between the alignment-space discrepancy and the downstream prediction discrepancy, we consider a local stability condition on the task-relevant feature manifold. Let $q(P_{i,b,j})={ẑ}_{i,b,j}$ denote the normalized alignment representation defined in Equations (14)–(17), and let $r(P_{i,b,j})$ denote the prediction associated with the corresponding local decoder region. We assume that, within the local feature neighborhood encountered during training, there exists a constant $C_{r}>0$ such that

(20) $${\left\|r(P_{1,b,j})-r(P_{2,b,j})\right\|}_{2}\leq C_{r}{\left\|{ẑ}_{1,b,j}-{ẑ}_{2,b,j}\right\|}_{2}$$

This local Lipschitz condition means that small changes in the normalized representation lead to proportionally bounded changes in the corresponding prediction, with $C_{r}$ characterizing the local sensitivity of the prediction mapping. Based on this condition, we define the region-weighted cross-view prediction discrepancy as:

(21) $$D_{pred}=\frac{1}{B}\sum_{b=1}^{B}\frac{\sum_{j=1}^{N}\omega_{b,j}{\left\|r(P_{1,b,j})-r(P_{2,b,j})\right\|}_{2}^{2}}{\sum_{j=1}^{N}\omega_{b,j}+\epsilon }\;\leq C_{r}^{2}\frac{1}{B}\sum_{b=1}^{B}\frac{\sum_{j=1}^{N}\omega_{b,j}{\left\|{ẑ}_{1,b,j}-{ẑ}_{2,b,j}\right\|}_{2}^{2}}{\sum_{j=1}^{N}\omega_{b,j}+\epsilon }\;=C_{r}^{2}L_{rpal}$$

We further consider the perturbation-induced component of the pseudo-label supervision discrepancy while holding the pseudo-label target fixed. Let ${\tilde{y}}_{b,j}$ denote a fixed pseudo-label associated with the corresponding region, and assume that the segmentation loss $l(p,\tilde{y})$ is locally $L_{l}$-Lipschitz with respect to the prediction p, meaning that local changes in the prediction induce proportionally bounded changes in the loss. The region-weighted difference in pseudo-label supervision between two perturbed views then satisfies:

(22) $$D_{sup}=\frac{1}{B}\sum_{b=1}^{B}\frac{\sum_{j=1}^{N}\omega_{b,j}\left|l(r(P_{1,b,j}),{\tilde{y}}_{b,j})-l(r(P_{2,b,j}),{\tilde{y}}_{b,j})\right|}{\sum_{j=1}^{N}\omega_{b,j}+\epsilon }\leq L_{l}C_{r}\sqrt{L_{rpal}}$$

Equation (22) shows that, under the stated local stability assumptions, reducing the RPAL discrepancy tightens an upper bound on the perturbation-induced variation in pseudo-label supervision for a fixed pseudo-label target. Importantly, this result does not imply that RPAL can correct an intrinsically incorrect pseudo-label or eliminate discrepancies caused by different pseudo-label targets. Instead, it characterizes how RPAL can limit the additional cross-view variation introduced by heterogeneous perturbations during pseudo-supervised training. 

### 3.3. Overall Objective 

The final training objective of RAHP combines the hybrid perturbation objective defined in Equation (11) with the proposed representation alignment loss:

(23) $$L_{total}=\lambda_{hybrid}L_{hybrid}+\lambda_{rpal}L_{rpal},$$

where $\lambda_{rpal}$ controls the contribution of the representation alignment loss. Since $L_{hybrid}$ already contains the supervised segmentation loss, the supervised term is not introduced separately in the final objective. This formulation enables labeled samples to provide direct segmentation supervision, while unlabeled samples contribute through image-level consistency, feature-level consistency, and patch-level representation alignment.

## 4. Experiments and Results

### 4.1. Datasets

In this study, we assessed the effectiveness of the proposed method using three publicly accessible datasets, ACDC [46], Synapse [47], and MMWHS [48].

**Automated cardiac diagnosis challenge dataset (ACDC):** The ACDC dataset comprises 200 annotated short-axis cardiac cine-MR volumes from 100 patients, each with segmentation masks for the left ventricle (LV), myocardium (Myo), and right ventricle (RV). According to the method in [15], the dataset was divided at the patient level into 70 patients for training, 10 for validation, and 20 for testing, corresponding to 140, 20, and 40 volumes, respectively. For semi-supervised training, 3 and 7 training patients were used as labeled data, corresponding to approximately 5% and 10% of the training patients. All slices from the same patient were assigned to the same subset.

**Synapse multi-organ segmentation dataset (Synapse):** The Synapse dataset contains 30 abdominal CT volumes with annotations for eight organs: the aorta, gallbladder, spleen, left kidney, right kidney, liver, pancreas, and stomach. The dataset was divided at the volume level into 18 volumes for training, 6 for validation, and 6 for testing. After preprocessing, the 18 training volumes contained 2211 two-dimensional slices. For semi-supervised training, 110 and 221 training slices were selected as labeled data for the 5% and 10% settings, respectively, while the remaining training slices were treated as unlabeled data.

**Multi-Modality Whole Heart Segmentation Dataset (MMWHS):** The MMWHS dataset provides whole-heart CT and MRI scans with pixel-level annotations for seven anatomical structures, including the left ventricle, right ventricle, left atrium, right atrium, myocardium, ascending aorta, and pulmonary artery. The dataset contains significant variations in imaging appearance, organ shape, and boundary quality, which introduce additional challenges for multi-organ segmentation. In this study, we used the 20 CT volumes and divided them at the volume level into 12 volumes for training, 4 for validation, and 4 for testing. After preprocessing, the 12 training volumes contained 3245 two-dimensional slices. For semi-supervised training, 162 and 324 training slices were selected as labeled data for the 5% and 10% settings, respectively, while the remaining training slices were treated as unlabeled data.

The labeled/unlabeled partition was defined at the patient level for ACDC and at the 2D slice level within the training volumes for Synapse and MMWHS. This difference reflects the labeling units used in our implementation: ACDC selects complete training patients as labeled cases, whereas the labeled-data ratios for Synapse and MMWHS are defined over the 2D training-slice pools. Consequently, labeled and unlabeled slices in Synapse and MMWHS may originate from the same training volume and may exhibit intra-volume correlation. This intra-volume correlation may reduce the independence between the labeled and unlabeled training subsets and may make their image distributions more similar than under a strictly patient-level labeled/unlabeled split. Therefore, the Synapse and MMWHS settings should be interpreted as slice-level limited-label scenarios rather than patient-level annotation-scarcity settings. For all three datasets, the training, validation, and test sets were separated at the patient or volume level.

### 4.2. Implementation Details

All experiments were conducted using PyTorch 2.1.0 on a single NVIDIA GeForce RTX 4090 GPU (NVIDIA Corporation, Santa Clara, CA, USA). For each iteration, the mini-batch size was set to 16, consisting of eight labeled and eight unlabeled two-dimensional slices. For all datasets, the pseudo-label confidence threshold was fixed a priori at 0.95 for the main experiments and was not selected using the validation set. The sensitivity analysis reported in Section 4.4.4 was conducted to evaluate the robustness of this predefined setting rather than to select the threshold, and the results show a favorable overall trade-off across the evaluated datasets. The weighting factors in the overall objective were fixed during training, where $\lambda_{hybrid}$ controlled the hybrid perturbation loss and $\lambda_{rpal}$ controlled the representation alignment loss, and both were set to 1. Inside the hybrid term, the image-level and feature-level perturbations were weighted by $\lambda_{img}=0.25$ and $\lambda_{feat}=0.5$. The model was optimized using Stochastic Gradient Descent with an initial learning rate of 0.01, a momentum of 0.9, and a weight decay of 0.0001. During training, the model parameters were periodically saved and evaluated on the validation set, and the checkpoint that achieved the best validation performance was used for final testing. To evaluate segmentation performance, we used the Dice Coefficient (DSC), Jaccard Index, 95% Hausdorff Distance (HD95), and Average Surface Distance (ASD).

All comparison methods were rerun using the same predefined dataset splits, labeled subsets, preprocessing pipeline, input resolution, training budget, batch size, and evaluation protocol. Model selection and any remaining hyperparameter adjustment were performed using only the validation set, and the test set was not used for model or hyperparameter selection. Publicly available implementations corresponding to the cited methods were used whenever possible. When no explicit release version was provided, the downloaded public code snapshot was used. Specifically, CPS was implemented with dual U-Net branches, while FixMatch and UniMatch used U-Net backbones. CTCT and S4CVNet retained their original U-Net/Swin-Unet dual-architecture designs, and ICL retained its Swin-Unet backbone. BCP used the original U-Net-based BCP_net, DiffRect retained its U-Net and UNet_LDMV2 components, and CFCT retained its dual U-Net architecture with clustering and segmentation heads. For architecture-agnostic methods, a common U-Net backbone was adopted to reduce architectural confounding, whereas architecture-dependent components were preserved when they were intrinsic to the original method formulation. The corresponding backbone configurations and method-specific components are summarized in Table 2. Nevertheless, architecture-specific modules, implementation choices, and method-specific training configurations may still affect absolute performance, and these effects cannot be completely eliminated in comparisons across methods with intrinsically different designs. Therefore, the reported results should be interpreted as comparisons under a substantially standardized experimental protocol rather than as completely architecture-isolated comparisons. To improve the reproducibility of the experimental evaluation, all main comparison experiments and ablation studies, including the component-wise, patch-size, gating-strategy, and hyperparameter-sensitivity analyses, were independently repeated three times using random seeds 0, 1, and 2. The dataset partitions and labeled subsets were kept fixed across all runs. Unless otherwise stated, the quantitative results are reported as the mean and standard deviation over the three independent runs. These summary statistics are used to characterize run-to-run variability. Given the limited number of independent runs, we do not perform formal hypothesis testing; all reported differences, rankings, and references to improvement are interpreted descriptively in terms of mean performance and are not presented as statistically significant effects.

In the RPAL module, decoder feature maps at an effective output stride of 4 were used for representation alignment. Each feature map contains 64 channels and is divided into non-overlapping patches of size 8 × 8. Spatial averaging within each patch produces a 64-dimensional patch representation. The token encoder was implemented as a two-layer multilayer perceptron consisting of a 64-to-128 linear layer, followed by a ReLU activation and a 128-to-64 linear layer, yielding a 64-dimensional embedding. In parallel, the gating branch consisted of a 64-to-32 linear layer, a ReLU activation, and a 32-to-1 linear layer followed by a sigmoid function to generate a scalar importance weight for each patch. The token encoder and gating branch were shared between the two sub-networks to maintain symmetric processing during representation alignment. All patch embeddings were L2-normalized before computing the alignment loss. The complete projection and gating module contains 18,689 trainable parameters (approximately 0.0187 M).

### 4.3. Results

We compared our proposed method with nine representative semi-supervised segmentation methods on the ACDC, Synapse, and MMWHS datasets. The compared methods include CPS [14], FixMatch [13], CTCT [15], S4CVNet [49], ICL [50], BCP [21], DiffRect [51], UniMatch [18], and CFCT [52].

#### 4.3.1. Results on ACDC

Table 3 reports the results on the ACDC dataset under the 5% and 10% labeled data settings. With 5% labeled data, most methods show noticeable performance degradation, while RAHP achieves the best mean results across all four metrics. RAHP obtains a DSC of 88.85%, a Jaccard score of 80.38%, an ASD of 0.65, and an HD95 of 1.67. Compared with CFCT, which achieves the second-best DSC, RAHP improves DSC by 1.27 percentage points. In addition, RAHP reduces HD95 by 0.09 mm compared with BCP, which obtains the second-lowest HD95. The improvement in boundary-related metrics suggests that the proposed representation alignment helps maintain more consistent local feature representations under limited supervision, particularly for structures with ambiguous boundaries. Compared with other methods, RAHP shows clear improvements in region overlap measurements and produces smaller boundary errors, indicating that the method can form stable predictions even with very limited labeled data. When the labeled portion increases to 10%, all methods improve to varying degrees. RAHP continues to yield the best overall mean performance with a DSC of 90.11%, a Jaccard score of 82.21%, and an HD95 of 1.70, while maintaining competitive ASD performance of 0.62. These results show that RAHP is able to make effective use of unlabeled samples and achieve favorable performance in both region accuracy and boundary precision.

**Figure 3:**
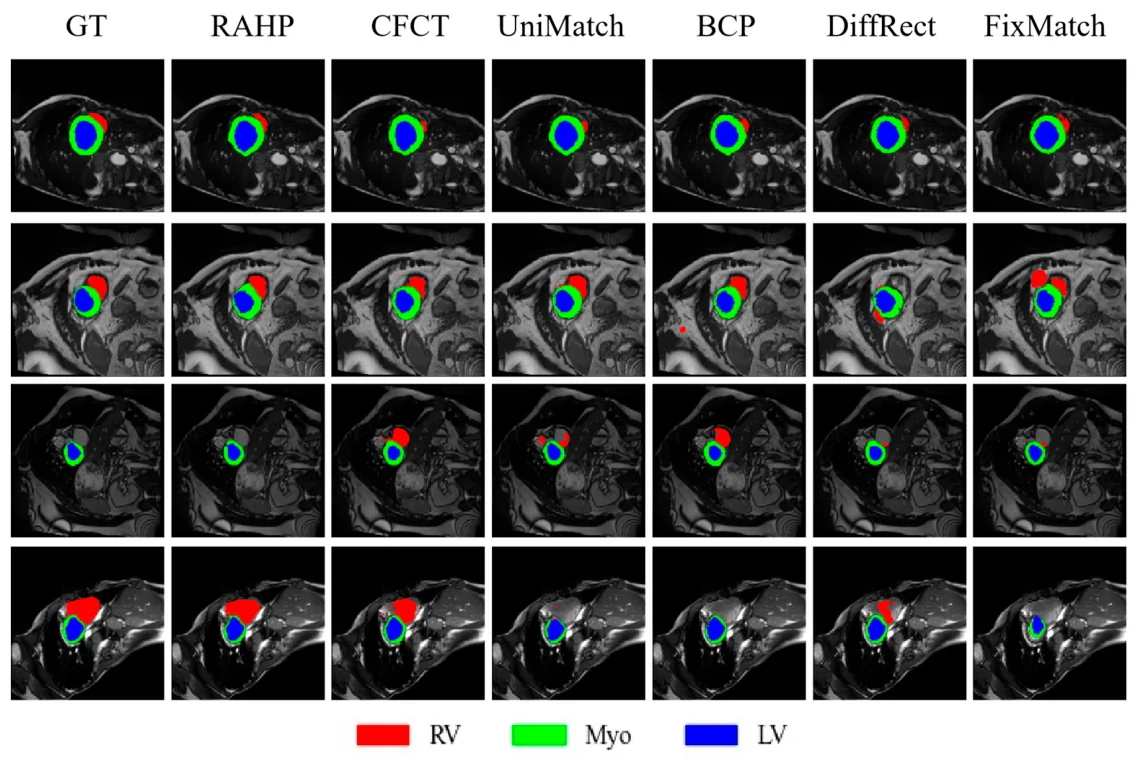
Visualization comparison of different methods on ACDC. The first two rows correspond to the setting with 3 labeled training patients (approximately 5%), while the last two rows correspond to the setting with 7 labeled training patients (10%).

Figure 3 illustrates the visual results of different segmentation methods on the ACDC dataset. The top two rows correspond to the setting with 5% labeled data, while the bottom two rows present the results obtained with 10% labeled data. From the visual results, it is evident that RAHP produces predictions that align more closely with the ground truth in both region coverage and boundary delineation. RAHP segments challenging structures such as the right ventricle and myocardium more accurately, even when these regions exhibit weak contrast and ambiguous boundaries, while other methods often produce fragmented regions or partially miss the target structures. Moreover, RAHP yields smoother contours and fewer false-positive regions under limited labeled data.

#### 4.3.2. Results on Synapse

The comparison on the Synapse dataset is presented in Table 4. Under the 5% labeled setting, the performance gap between different methods becomes more apparent due to the large anatomical variations in abdominal organs. RAHP achieves the best mean results across all four metrics, reaching a DSC of 81.03%, a Jaccard score of 70.68%, an ASD of 8.35, and an HD95 of 27.55. These results indicate that maintaining stable intermediate representations is particularly beneficial for multi-organ segmentation, where different anatomical structures often exhibit large variations in appearance and spatial relationships. With 10% labeled data, RAHP again obtains the best overall mean performance, achieving a DSC of 82.84%, a Jaccard score of 72.14%, an ASD of 6.32, and an HD95 of 25.42. Relative to UniMatch, RAHP obtains numerically better mean values across all four evaluation metrics. These numerical differences relative to UniMatch are generally larger than those observed on ACDC, suggesting that the proposed hybrid perturbation and region alignment strategies may be particularly beneficial in the evaluated multi-class setting.

**Figure 4:**
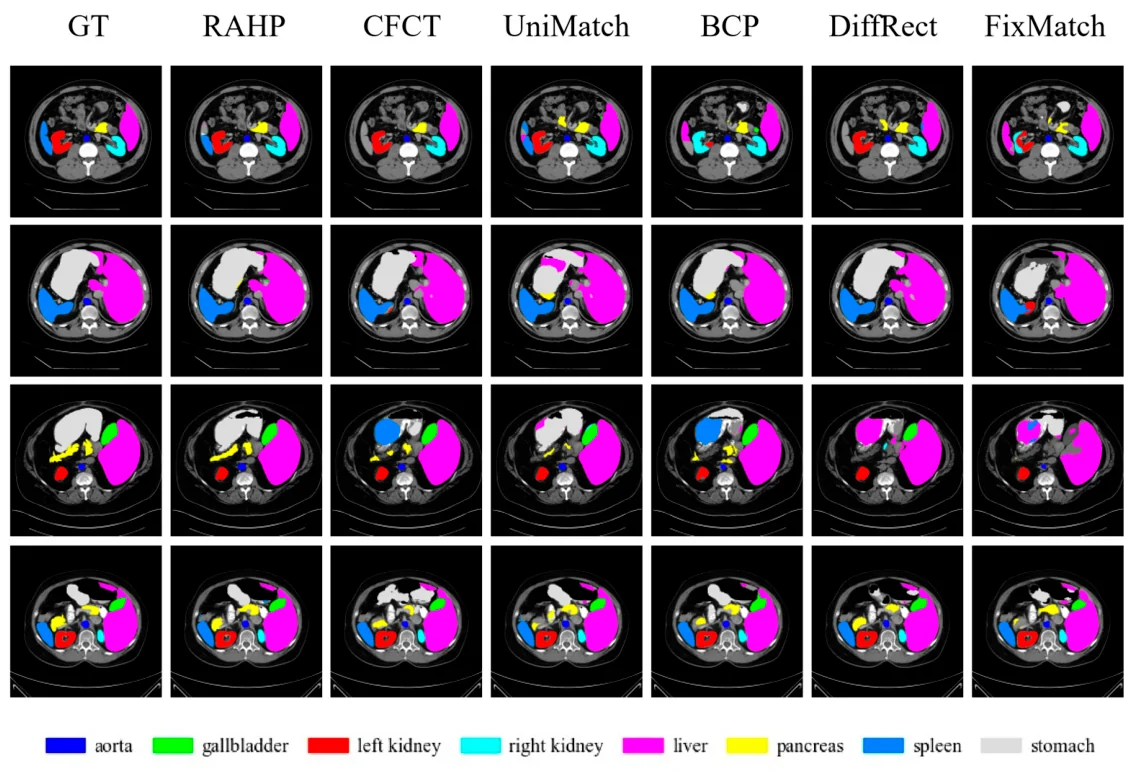
Visualization comparison of different methods on Synapse. The first two rows correspond to the setting with 5% labeled training slices, while the last two rows correspond to the setting with 10% labeled training slices.

Figure 4 shows the segmentation visualizations of different methods on the Synapse dataset. RAHP produces more complete organ regions and clearer separation between adjacent abdominal structures than the competing methods. Several baselines exhibit fragmented predictions or incorrect merging when organs are closely located or have similar intensity distributions. For example, in the third row of Figure 4, methods such as CFCT and UniMatch show discontinuous segmentation of the pancreas and partial confusion between the stomach and surrounding tissues. In contrast, RAHP maintains a more coherent pancreas region and preserves clearer boundaries between neighboring organs.

#### 4.3.3. Results on MMWHS

Table 5 summarizes the results on the MMWHS dataset. With 5% labeled samples, RAHP achieves the best mean performance among all comparison methods, with a DSC of 90.54%, a Jaccard score of 82.95%, an ASD of 3.35, and an HD95 of 9.17. These results indicate that RAHP can produce stable predictions on cardiac structures with diverse shapes and scales. When the number of labeled samples increases to 10%, RAHP continues to achieve the best mean results across all four metrics. The method reaches a DSC of 91.19%, a Jaccard score of 83.58%, an ASD of 2.75, and an HD95 of 6.74. Overall, RAHP achieves favorable mean performance across all four metrics, suggesting that the proposed hybrid perturbation and region-aware alignment mechanisms contribute to improved segmentation performance on complex cardiac anatomy.

**Figure 5:**
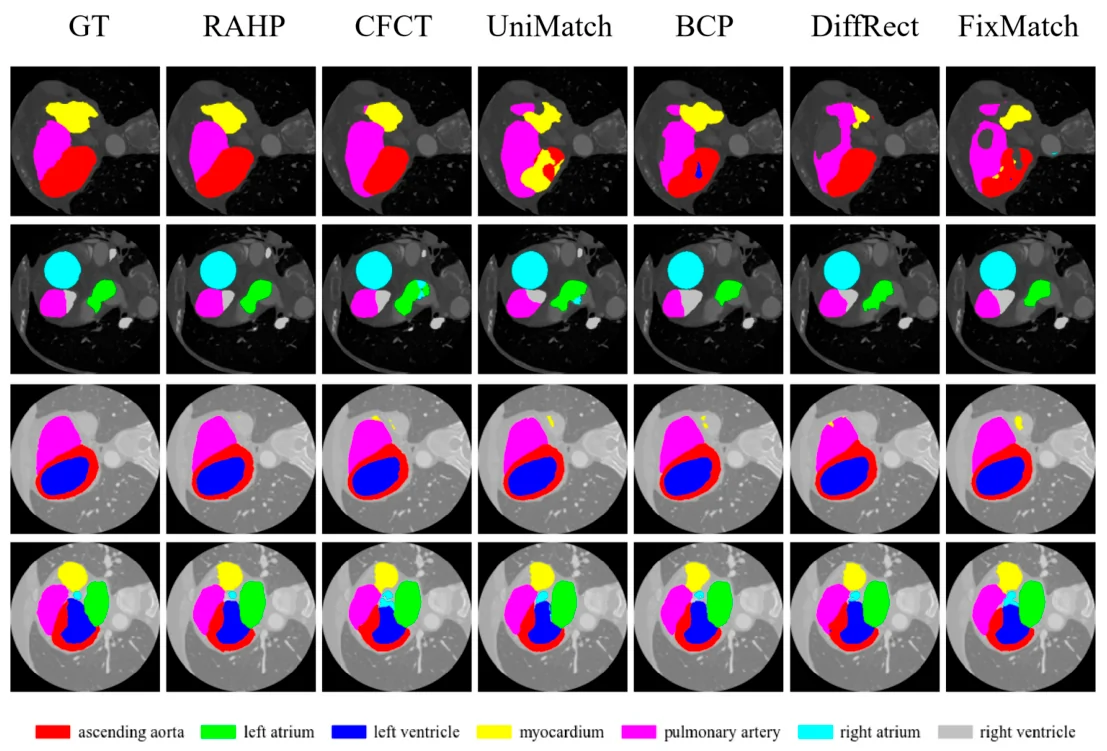
Visualization comparison of different methods on MMWHS. The first two rows correspond to the setting with 5% labeled training slices, while the last two rows correspond to the setting with 10% labeled training slices.

Figure 5 presents representative visual segmentation results on the MMWHS dataset. RAHP yields more accurate and stable segmentations for both large cardiac chambers and thin anatomical structures, while several baseline methods exhibit boundary leakage or partial misclassification between adjacent regions, such as the left ventricle and myocardium. These visual observations are consistent with the boundary-related trends reported in Table 5.

The visual examples in Figure 3, Figure 4 and Figure 5 are intended to provide qualitative illustrations of typical segmentation behavior rather than to establish general performance. The overall boundary accuracy is evaluated quantitatively using ASD and HD95 over the complete test sets, as reported in Table 3, Table 4 and Table 5 with mean and standard deviation over three independent runs. These aggregate results provide the primary quantitative evidence for the boundary-related performance of RAHP, while the visual examples complement the numerical evaluation by illustrating typical error patterns.

### 4.4. Ablation Study

This section systematically analyzes the contribution of each component in RAHP, including perturbation strategies, RPAL design, and patch-level configurations.

#### 4.4.1. Effectiveness of the Proposed Methods

We first examine how the hybrid perturbation strategy and the RPAL module contribute to the overall performance of RAHP. As shown in Table 6, the ablation settings progressively introduce cross-teaching, image-level perturbation, feature-level perturbation, and RPAL, allowing the contribution and interaction of these components to be evaluated under limited supervision.

On the ACDC dataset with 5% labeled data, cross-teaching achieves 86.92% DSC. Adding image-level strong perturbation improves the DSC to 88.18%, whereas using feature-level perturbation alone decreases the DSC to 85.45%, which is 1.47 percentage points below the cross-teaching baseline. This result suggests that feature perturbation by itself may introduce excessive representation variation without an additional stabilizing constraint. When image- and feature-level perturbations are combined within the hybrid perturbation framework, the DSC increases to 88.38%. On this basis, introducing RPAL further increases the DSC from 88.38% to 88.85% (+0.47 percentage points) and Jaccard from 80.30% to 80.38%, while reducing ASD from 0.73 to 0.65 and HD95 from 2.23 to 1.67. Although these boundary metrics are not the absolute best among all individual ablation settings, they remain close to the best observed values while being achieved together with the highest DSC and Jaccard in the complete configuration.

On Synapse, feature-level perturbation alone similarly decreases the DSC from 73.87% for cross teaching to 72.93%, whereas the complete hybrid perturbation setting reaches 80.42%. Adding RPAL further increases the DSC to 81.03% (+0.61 percentage points), reduces ASD from 8.68 to 8.35, and decreases HD95 from 29.64 to 27.55. Jaccard shows a slight decrease from 71.13% to 70.68%. These results show descriptive improvements in DSC, ASD, and HD95 on Synapse, while Jaccard decreases slightly. Therefore, the effect of RPAL is metric-dependent rather than uniformly positive.

Overall, these results show that increasing perturbation diversity alone does not necessarily improve segmentation performance, as evidenced by the degradation caused by feature perturbation when used independently. The direct comparison between the hybrid setting with and without RPAL further isolates the contribution of representation alignment. In terms of mean values, RPAL improves DSC and boundary-related metrics on both ACDC and Synapse, while its effect varies across datasets and metrics. These findings suggest that RPAL is particularly useful for constraining perturbation-induced representation variation, while its quantitative benefit remains dependent on the specific dataset and evaluation measure.

#### 4.4.2. Ablation on Patch Size

We evaluated the sensitivity of RPAL to the patch size p on ACDC under the 5% labeled setting. Three patch sizes, p∈{4,8,16} were evaluated over three independent runs. As shown in Table 7, p=8 achieves the highest mean DSC (88.85%) and Jaccard (80.38%), as well as the lowest HD95 (1.67). A smaller patch size of 4 yields a slightly lower ASD (0.61 versus 0.65) but also a lower DSC and a higher HD95. Increasing the patch size to 16 results in a further decrease in overlap accuracy. Overall, p=8 provides the most favorable balance between overlap accuracy and boundary performance, and is therefore adopted as the default setting for RPAL.

#### 4.4.3. Ablation on Gate Design

We further evaluated the effect of the gating strategy in RPAL using three independent runs on ACDC with 5% labeled data. As shown in Table 8, the sigmoid gate achieves the highest mean DSC (88.85%) and the lowest ASD (0.65) and HD95 (1.67). The softmax strategy obtains a slightly higher mean Jaccard score (80.46% versus 80.38%), but produces substantially larger ASD and HD95 values. Removing the gating mechanism further reduces the DSC to 88.19%. These results suggest that the sigmoid gate provides a favorable overall trade-off between overlap accuracy and boundary quality and is therefore used in the final configuration.

#### 4.4.4. Hyperparameter Sensitivity Analysis

We further investigated the sensitivity of RAHP to two important RPAL hyperparameters: the embedding dimension *D* and the representation alignment weight $\lambda_{rpal}$. The experiments were conducted on ACDC with 5% labeled data, while varying one hyperparameter at a time and keeping the remaining settings fixed. As shown in Table 9, the embedding dimension *D* = 64 achieves the best overall performance, with 88.85% DSC, 80.38% Jaccard, 0.65 ASD, and 1.67 HD95. Reducing *D* to 32 leads to a clear decrease in overlap accuracy, whereas increasing it to 128 does not provide further improvement. For the RPAL loss-weight analysis, $\lambda_{hybrid}$ was fixed to 1, and $\lambda_{rpal}$ was varied while all other settings were kept unchanged. As shown in Table 9, $\lambda_{rpal}=1$ provides the most favorable overall performance. A smaller weight of 0.5 produces slightly lower segmentation accuracy, while increasing the weight to 2 results in a larger degradation, particularly in HD95. This suggests that excessively emphasizing representation alignment may interfere with the balance between segmentation supervision and feature regularization. Based on these results, *D* = 64 and $\lambda_{rpal}=1$ are used as the default settings. 

Since the pseudo-label confidence threshold τ directly determines which unlabeled predictions are retained for consistency supervision, we additionally evaluated τ∈{0.85,0.95,0.98} on all three datasets under the 5% labeled setting. As shown in Table 10, τ=0.95 provides the most favorable overall mean performance across the evaluated metrics on ACDC, Synapse, and MMWHS. For example, on ACDC, the DSC increases from 87.39% at τ=0.85 to 88.85% at τ=0.95, while HD95 decreases from 1.97 to 1.67. Increasing the threshold further to 0.98 slightly reduces the DSC to 88.39%. Similar trends are observed on Synapse and MMWHS. The results indicate that the performance is sensitive to the confidence threshold but remains stable within the evaluated range. A relatively low threshold may admit less reliable pseudo labels, whereas an excessively high threshold may discard useful unlabeled predictions. The intermediate value τ=0.95 provides a consistent trade-off across the three datasets. These retrospective sensitivity results support the robustness of the predefined threshold used in the main experiments.

#### 4.4.5. Visualization of Region-Aware Patch Responses

**Figure 6:**
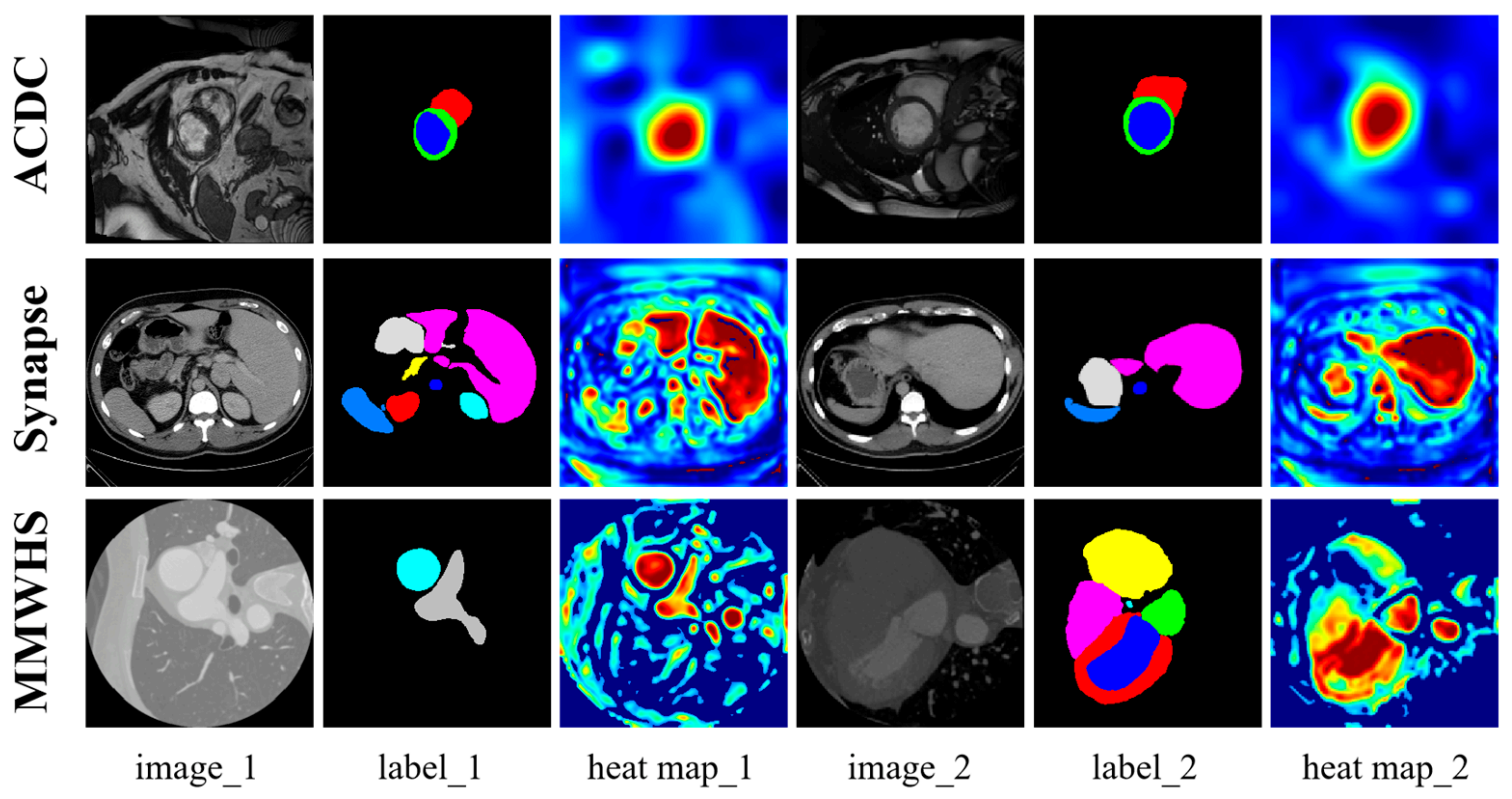
Visualization of region-aware patch response heatmaps derived from the RPAL weighting mechanism on representative test samples. Warmer colors in the heatmaps indicate higher region-aware response weights, whereas cooler colors indicate lower responses; colors in the label maps distinguish different anatomical classes.

To further analyze the effect of the proposed RPAL on feature learning, we visualize the region-aware patch responses on representative test samples. The decoder feature map at stride 4 is divided into non-overlapping patches, and patch-wise importance weights are computed by the region-aware weighting mechanism in RPAL. These weights are then upsampled to the original image resolution to generate heatmaps, which reflect the spatial distribution of region-level responses guided by RPAL. As illustrated in Figure 6, the high-response regions show spatial correspondence with the target structures indicated by the ground-truth labels, while most background areas exhibit relatively low responses. Enhanced responses can also be observed in organ interiors and boundary-adjacent regions. These response patterns suggest that the learned region-aware weights tend to emphasize anatomically relevant regions during intermediate feature alignment and are consistent with the intended role of RPAL in promoting more spatially focused representations under perturbations.

### 4.5. Computational Complexity Analysis

To evaluate the computational efficiency of the proposed method, the numbers of trainable parameters and multiply–accumulate operations (MACs) were measured using a single-channel input of size 256 × 256. Inference latency was measured on the same NVIDIA RTX 4090 GPU with a batch size of 1. Since RPAL is used only during training, only a single trained U-Net is retained for inference, without the RPAL module or the second cross-teaching branch.

Table 11 summarizes the computational complexity and inference efficiency of the cross-teaching baseline and RAHP under the ACDC configuration. The cross-teaching framework contains two U-Net branches during training, resulting in 3.6275 M trainable parameters. Incorporating RPAL increases the total number of training parameters to 3.6462 M. Therefore, RPAL introduces only 0.0187 M additional parameters, corresponding to an increase of approximately 0.52%. The RPAL alignment operation itself requires only 0.0024 G additional MACs. Importantly, RPAL is employed only as a training-time representation constraint and does not participate in inference. The second cross-teaching branch is also not required during testing. Consequently, the final inference model consists of a single U-Net with 1.8138 M parameters and 3.0033 G MACs for a 256 × 256 two-dimensional input. The measured inference latency is 2.26 ± 0.72 ms per slice on a single NVIDIA RTX 4090 GPU. These results indicate that RPAL introduces only a small additional parameter and alignment-computation overhead during training while retaining the same inference architecture and theoretical inference complexity as the cross-teaching baseline.

## 5. Discussion and Conclusions

Consistency regularization has become an effective paradigm for semi-supervised medical image segmentation by encouraging prediction invariance under different perturbations. Existing studies have demonstrated that diverse perturbation strategies, including network-level, image-level, and feature-level perturbations, can improve the utilization of unlabeled data by providing complementary training views. However, increasing perturbation diversity may also introduce additional representation variations, making it difficult for networks to maintain stable intermediate features across different views. With this consideration, we propose RAHP, a representation stabilization framework that integrates multi-level perturbation learning with explicit representation alignment. Specifically, network-level, image-level, and feature-level perturbations are employed to generate diverse training views, while RPAL further constrains intermediate representations across different views. This combination provides complementary consistency constraints at different levels while stabilizing feature representations under diverse perturbations, thereby improving the model’s ability to exploit unlabeled data. Experimental results on three publicly available medical image datasets show favorable mean segmentation performance under the evaluated limited-label settings. The results indicate that combining hybrid perturbation learning with representation alignment can improve the utilization of unlabeled data, although the magnitude of the improvement varies across datasets and evaluation metrics.

Despite the promising results, several limitations remain in the current study. First, the proposed framework relies mainly on consistency regularization and uses a fixed confidence threshold for pseudo-label filtering. More adaptive pseudo-label selection strategies may further improve the reliability of unlabeled supervision. Second, the experiments were conducted on three public benchmark datasets under the 5% and 10% labeled settings, and external multi-center or clinical validation has not yet been performed. The current implementation also operates on two-dimensional slices and therefore does not explicitly exploit inter-slice or volumetric contextual information. Moreover, for Synapse and MMWHS, the labeled and unlabeled subsets are constructed at the slice level within the training volumes. As a result, labeled and unlabeled slices may originate from the same volume and share intra-volume anatomical and imaging characteristics, which may reduce the independence between the two training subsets. Although the training, validation, and test partitions remain strictly separated at the patient/volume level and therefore do not introduce evaluation-set leakage, this slice-level sampling protocol represents a less restrictive setting than patient-level labeled/unlabeled separation. Future evaluation under patient-level annotation scarcity and on external multi-center datasets will be important for assessing the generalizability of RAHP more comprehensively. In addition, the present evaluation focuses on single-modality MRI or CT segmentation, while cross-modal information fusion and acquisition-specific variations are not explicitly considered. The dual-network training framework and RPAL also introduce additional computation during training, although only a single U-Net is retained for inference, as discussed in Section 4.5. Future work will investigate more adaptive pseudo-labeling strategies, three-dimensional modeling, multimodal extensions, broader annotation regimes, and validation on more diverse and multi-center datasets.

## Figures and Tables

**Table 1:** Details of Perturbation Methods and Parameter Configurations in RAHP.

Perturbation Type	Perturbation Method	Description	Parameter Settings
Weak Perturbation	Cropping	Randomly crops a portion of the image	Random crop (10–20%)
	Flipping	Randomly flips the image horizontally or vertically	Random selection (50% probability)
	Rotation	Rotates the image by a random angle	Random angle (−20° to 20°)
Strong Perturbation	Color Jitter	Changes brightness, contrast, saturation, and hue	Brightness (0.5 to 1.25), Contrast (0.5 to 1.25), Saturation (0.5 to 1.25), Hue 0.5
	Random Blur	Applies one of Gaussian, Average, or Median blur randomly	Gaussian (sigma 0.1–2.0), Average (random radius), Median (random radius)
	CutMix	Crops a patch from the image and replaces it with a patch from another image	Random crop size and position
Feature Perturbation	Channel Dropout	Randomly drops out channels from intermediate feature maps	dropout probability 50%

**Table 2:** Backbone configurations and method-specific components of the evaluated methods.

Method	Backbone/Main Network	Method-Specific Component
CPS	Dual U-Net	Cross pseudo supervision between two independently initialized networks
FixMatch	U-Net	Weak-to-strong consistency with an EMA teacher
CTCT	U-Net + Swin-Unet	CNN–Transformer cross teaching
S4CVNet	U-Net + Swin-Unet	CNN–Transformer cross pseudo supervision with an EMA teacher
ICL	Swin-Unet	Swin Transformer-based segmentation architecture
BCP	U-Net-based BCP_net	EMA teacher with bidirectional copy-paste
DiffRect	U-Net + UNet_LDMV2	Latent diffusion-based pseudo-label rectification
UniMatch	U-Net	Dual strong-view consistency with CutMix and feature perturbation
CFCT	Dual U-Net with clustering and segmentation heads	Cluster-based dual-branch cross teaching
RAHP (Ours)	Dual U-Net	Hybrid network-/image-/feature-level perturbation with region-aware patch alignment (RPAL)

**Table 3:** Comparison results of the proposed method on the ACDC dataset. Results are reported as mean ± standard deviation over three independent runs. Bold values indicate the best mean result for each metric under each setting.

Method	Labels Used		Metrics			
	Labeled	Unlabeled	DSC	Jaccard	ASD	HD95
CPS	5%	95%	61.48 ± 0.89	50.82 ± 1.49	1.76 ± 0.37	27.27 ± 1.18
FixMatch			63.43 ± 1.26	53.45 ± 1.65	4.72 ± 0.27	27.93 ± 1.97
CTCT			71.80 ± 1.20	63.84 ± 1.38	1.05 ± 0.37	16.78 ± 1.31
S4CVNet			76.46 ± 0.73	63.00 ± 0.75	1.23 ± 0.14	20.39 ± 3.21
ICL			81.03 ± 1.10	70.04 ± 0.91	2.08 ± 0.67	7.66 ± 1.16
BCP			87.00 ± 0.32	79.91 ± 0.46	0.74 ± 0.08	1.76 ± 1.81
DiffRect			83.13 ± 0.59	76.00 ± 1.03	1.46 ± 0.13	5.97 ± 0.88
UniMatch			86.29 ± 0.21	79.20 ± 0.69	0.71 ± 0.11	3.69 ± 0.36
CFCT			87.58 ± 0.23	77.66 ± 0.51	0.72 ± 0.26	4.32 ± 0.29
RAHP			**88.85 ± 1.04**	**80.38 ± 0.38**	**0.65 ± 0.05**	**1.67 ± 0.21**
CPS	10%	90%	85.03 ± 0.64	75.17 ± 0.96	2.96 ± 0.76	10.45 ± 1.68
FixMatch			88.48 ± 0.84	79.08 ± 0.48	0.64 ± 0.20	3.21 ± 0.49
CTCT			87.17 ± 0.72	76.01 ± 0.74	2.46 ± 0.24	9.97 ± 0.72
S4CVNet			86.79 ± 0.45	78.28 ± 0.21	1.36 ± 0.35	4.90 ± 0.44
ICL			88.38 ± 0.66	80.05 ± 0.37	0.65 ± 0.21	2.11 ± 1.03
BCP			88.93 ± 1.01	81.01 ± 0.61	1.11 ± 0.07	3.64 ± 0.46
DiffRect			88.68 ± 1.41	80.42 ± 1.22	0.83 ± 0.06	2.41 ± 0.46
UniMatch			88.27 ± 0.72	79.97 ± 0.58	**0.** **5** **7 ± 0.** **2** **8**	2.85 ± 0.12
CFCT			88.93 ± 0.42	80.48 ± 0.53	0.63 ± 0.06	1.91 ± 0.25
RAHP			**90.11 ± 0.45**	**82.21 ± 0.78**	0.62 ± 0.16	**1.70 ± 0.42**

**Table 4:** The segmentation results of different competing methods on the Synapse dataset. Results are reported as mean ± standard deviation over three independent runs. Bold values indicate the best mean result for each metric under each setting.

Method	Labels Used		Metrics			
	Labeled	Unlabeled	DSC	Jaccard	ASD	HD95
CPS	5%	95%	68.38 ± 1.04	53.86 ± 0.73	20.56 ± 0.88	63.68 ± 2.09
FixMatch			63.83 ± 0.55	50.54 ± 1.27	21.01 ± 0.60	52.34 ± 1.67
CTCT			69.57 ± 1.07	53.94 ± 0.42	18.31 ± 0.81	58.22 ± 3.45
S4CVNet			70.87 ± 0.73	57.59 ± 0.67	20.80 ± 0.77	64.89 ± 1.37
ICL			70.03 ± 0.89	57.33 ± 0.47	20.46 ± 1.03	55.94 ± 1.28
BCP			76.89 ± 0.35	66.39 ± 0.59	13.16 ± 1.27	45.22 ± 2.20
DiffRect			72.73 ± 1.33	61.56 ± 0.46	20.38 ± 0.46	58.66 ± 3.03
UniMatch			76.34 ± 0.71	66.12 ± 0.96	16.54 ± 0.61	53.14 ± 3.29
CFCT			77.66 ± 1.46	65.20 ± 0.37	10.05 ± 1.27	40.65 ± 0.89
RAHP			**81.03 ± 0.99**	**70.68 ± 0.94**	**8.35 ± 1.10**	**27.55 ± 1.31**
CPS	10%	90%	67.80 ± 0.65	56.66 ± 0.47	9.10 ± 0.94	61.75 ± 4.24
FixMatch			69.70 ± 0.98	56.54 ± 1.12	8.67 ± 0.68	55.87 ± 1.30
CTCT			71.93 ± 0.63	56.14 ± 0.90	9.35 ± 0.50	31.71 ± 0.73
S4CVNet			72.78 ± 1.04	59.32 ± 0.43	9.64 ± 1.34	43.73 ± 4.44
ICL			72.79 ± 0.55	60.45 ± 0.58	10.54 ± 0.58	52.89 ± 1.99
BCP			77.31 ± 0.34	66.87 ± 0.47	9.37 ± 0.51	33.32 ± 1.53
DiffRect			77.14 ± 0.92	63.95 ± 0.83	12.04 ± 0.60	37.18 ± 1.56
UniMatch			77.71 ± 1.48	69.35 ± 0.55	10.62 ± 0.60	42.81 ± 0.92
CFCT			78.76 ± 0.40	69.34 ± 0.58	10.40 ± 1.11	38.47 ± 3.61
RAHP			**82.84 ± 0.36**	**72.14 ± 0.94**	**6.32 ± 0.34**	**25.42 ± 1.67**

**Table 5:** The segmentation results of different competing methods on the MMWHS dataset. Results are reported as mean ± standard deviation over three independent runs. Bold values indicate the best mean result for each metric under each setting.

Method	Labels Used		Metrics			
	Labeled	Unlabeled	DSC	Jaccard	ASD	HD95
CPS	5%	95%	85.74 ± 0.27	74.95 ± 0.43	9.01 ± 0.32	38.37 ± 2.70
FixMatch			87.46 ± 0.63	80.13 ± 0.45	5.30 ± 0.52	15.13 ± 0.73
CTCT			84.73 ± 0.71	72.56 ± 0.76	12.20 ± 1.00	44.53 ± 1.13
S4CVNet			86.34 ± 0.92	74.69 ± 0.91	7.61 ± 0.15	25.04 ± 1.64
ICL			90.45 ± 0.68	81.95 ± 0.43	3.98 ± 0.11	12.02 ± 1.55
BCP			89.23 ± 0.53	81.23 ± 1.13	6.50 ± 0.53	19.49 ± 1.88
DiffRect			87.32 ± 0.17	80.17 ± 0.33	3.83 ± 0.27	9.45 ± 0.40
UniMatch			89.33 ± 0.26	82.83 ± 0.30	4.20 ± 0.81	16.07 ± 1.07
CFCT			87.41 ± 0.66	81.25 ± 0.44	5.36 ± 0.90	14.04 ± 1.07
RAHP			**90.54 ± 0.43**	**82.95 ± 0.36**	**3.35 ± 0.22**	**9.17 ± 1.30**
CPS	10%	90%	86.72 ± 0.13	75.57 ± 0.14	8.70 ± 0.20	23.77 ± 1.83
FixMatch			89.54 ± 0.40	83.11 ± 0.39	4.32 ± 0.16	9.01 ± 1.73
CTCT			87.54 ± 0.11	78.02 ± 0.36	6.94 ± 0.10	22.11 ± 2.74
S4CVNet			87.45 ± 0.58	79.99 ± 0.51	5.00 ± 0.10	15.96 ± 2.87
ICL			89.00 ± 0.30	83.04 ± 0.36	3.45 ± 0.68	10.22 ± 0.83
BCP			89.37 ± 0.63	82.56 ± 0.92	5.62 ± 0.61	11.65 ± 0.93
DiffRect			88.89 ± 1.08	80.48 ± 0.27	3.61 ± 0.40	11.58 ± 1.32
UniMatch			90.05 ± 0.66	82.61 ± 0.34	3.74 ± 0.28	17.39 ± 0.19
CFCT			88.78 ± 0.61	81.44 ± 0.17	4.29 ± 0.64	6.83 ± 1.37
RAHP			**91.19 ± 0.67**	**8** **3** **.58 ± 0.83**	**2.75 ± 0.84**	**6.74 ± 1.02**

**Table 6:** Component-wise ablation study of RAHP on ACDC and Synapse datasets. Results are reported as mean ± standard deviation over three independent runs. Bold values indicate the best mean result for each metric within each dataset.

Dataset	Labeled	Cross Teaching	Strong Perturbation	Feature Perturbation	RPAL	DSC	Jaccard	ASD	HD95
ACDC	5%	✓				86.92 ± 0.68	78.27 ± 0.42	1.27 ± 0.07	4.43 ± 0.06
		✓	✓			88.18 ± 0.58	78.44 ± 0.66	**0.58 ± 0.10**	**1.65 ± 0.43**
		✓		✓		85.45 ± 0.83	74.09 ± 0.46	1.34 ± 0.18	4.42 ± 0.45
		✓	✓	✓		88.38 ± 0.61	80.30 ± 0.31	0.73 ± 0.10	2.23 ± 0.11
		✓	✓	✓	✓	**88.85 ± 1.04**	**80.38 ± 0.38**	0.65 ± 0.05	1.67 ± 0.21
Synapse	5%	✓				73.87 ± 0.68	60.84 ± 0.70	9.47 ± 0.74	46.01 ± 3.28
		✓	✓			77.52 ± 1.55	68.53 ± 0.90	10.18 ± 0.16	38.91 ± 3.06
		✓		✓		72.93 ± 0.40	59.82 ± 1.55	10.56 ± 0.43	51.98 ± 0.69
		✓	✓	✓		80.42 ± 1.03	**71.13 ± 0.74**	8.68 ± 0.31	29.64 ± 3.15
		✓	✓	✓	✓	**81.03 ± 0.99**	70.68 ± 0.94	**8.35 ± 1.10**	**27.55 ± 1.31**

**Table 7:** Ablation study on patch size selection for RPAL using the ACDC dataset with 5% labeled data. Results are reported as mean ± standard deviation over three independent runs. Bold values indicate the best mean result for each metric.

Patch Size	DSC	Jaccard	ASD	HD95
4	88.72 ± 1.38	80.21 ± 0.57	0.61 ± 0.08	2.09 ± 0.42
8	**88.85 ± 1.04**	**80.38 ± 0.38**	**0.65 ± 0.05**	**1.67 ± 0.21**
16	88.25 ± 0.98	79.47 ± 1.01	0.64 ± 0.03	1.79 ± 0.34

**Table 8:** Ablation study on the gating strategy of RPAL on ACDC with 5% labeled data. Results are reported as mean ± standard deviation over three independent runs. Bold values indicate the best mean result for each metric.

Gate Design	DSC	Jaccard	ASD	HD95
sigmoid gate	**88.85 ± 1.04**	**80.38 ± 0.38**	**0.65 ± 0.05**	**1.67 ± 0.21**
softmax across patches	88.77 ± 1.21	80.46 ± 0.72	0.82 ± 0.11	3.12 ± 0.43
no gate	88.19 ± 1.16	79.62 ± 0.58	0.76 ± 0.07	2.79 ± 0.30

**Table 9:** Sensitivity analysis of the embedding dimension and RPAL loss weight on ACDC with 5% labeled data. Results are reported as mean ± standard deviation over three independent runs. Bold values indicate the best mean result for each metric.

Hyperparameter	Value	DSC	Jaccard	ASD	HD95
Embedding dimension	32	87.24 ± 0.95	78.42 ± 0.41	0.81 ± 0.07	1.88 ± 0.18
	64	**88.85 ± 1.04**	**80.38 ± 0.38**	**0.65 ± 0.05**	**1.67 ± 0.21**
	128	88.15 ± 1.11	79.62 ± 0.60	0.70 ± 0.13	1.78 ± 0.32
RPAL weight	0.5	88.45 ± 1.45	80.00 ± 0.44	0.68 ± 0.04	1.71 ± 0.32
	1	**88.85 ± 1.04**	**80.38 ± 0.38**	**0.65 ± 0.05**	**1.67 ± 0.21**
	2	87.62 ± 1.25	79.15 ± 0.48	0.72 ± 0.08	2.62 ± 0.28

**Table 10:** Sensitivity analysis of the pseudo-label confidence threshold on ACDC, Synapse, and MMWHS under the 5% labeled setting. Results are reported as mean ± standard deviation over three independent runs. Bold values indicate the best mean result for each metric within each dataset.

Confidence Threshold	Dataset	DSC	Jaccard	ASD	HD95
0.85	ACDC	87.39 ± 1.96	78.88 ± 1.17	0.78 ± 0.15	1.97 ± 0.41
0.95		**88.85 ± 1.04**	**80.38 ± 0.38**	**0.65 ± 0.05**	**1.67 ± 0.21**
0.98		88.39 ± 1.28	79.87 ± 0.45	0.70 ± 0.04	1.73 ± 0.22
0.85	Synapse	80.63 ± 0.89	70.40 ± 0.99	9.18 ± 1.37	28.02 ± 1.21
0.95		**81.03 ± 0.99**	**70.68 ± 0.94**	**8.35 ± 1.10**	**27.55 ± 1.31**
0.98		80.54 ± 1.02	70.02 ± 0.97	9.01 ± 1.12	29.10 ± 1.46
0.85	MMWHS	90.29 ± 0.49	82.74 ± 0.32	3.69 ± 0.23	9.80 ± 1.11
0.95		**90.54 ± 0.43**	**82.95 ± 0.36**	**3.35 ± 0.22**	**9.17 ± 1.30**
0.98		89.91 ± 0.50	81.91 ± 0.44	3.94 ± 0.45	10.65 ± 1.51

**Table 11:** Computational complexity and inference efficiency. “–” indicates not applicable.

Method	Training Params (M)	Additional RPAL MACs (G)	Inference Params (M)	Inference MACs (G)	Inference Time (ms/slice)
Cross Teaching	3.6275	–	1.8138	3.0033	2.26 ± 0.72
RAHP	3.6462	0.0024	1.8138	3.0033	2.26 ± 0.72

## Data Availability

The datasets used in this study are publicly available from the ACDC Challenge (https://www.creatis.insa-lyon.fr/Challenge/acdc/databases.html), Synapse Multi-Atlas Abdomen Labeling Challenge (https://www.synapse.org/#!Synapse:syn3193805/wiki/217789), and MM-WHS Challenge repositories (https://zmiclab.github.io/zxh/0/mmwhs/data.html), accessed on 8 October 2026. No new datasets were generated in this study.
